# Brief Hints on Diseases of the Ear in Children*Paper delivered before Pennsylvania State Medical Society Meeting, in Philadelphia, May 17, 1894.

**Published:** 1894-08

**Authors:** Laurence Turnbull

**Affiliations:** Philadelphia, Pa.


BRIEF HINTS ON DISEASES OF THE EAR IN CHILDREN.*—The subject of Wharton's jelly in the ear of a child, as a rule, has been found in the premature infant, followed by irritation and a discharge. There are other cases where the labor is difficult and prolonged, and when the ear becomes filled with blood, meconium and the genital secretions of the mother, causing a muco-purulent discharge, which must be cleansed by an intelligent nurse, such as may be found in our hospitals, where instruction has been given to that effect. No inflammation has resulted from such treatment, nor has dermatitis ever followed the cleansing.

In the treatment of otalgia or earache in the new-born, no remedy has been found so successful as a two per cent. solution of cocaine, applied as hot as the patient can bear it; or heat alone—dry heat being preferred—as a bag of hot salt or hops. Should there be severe inflammation, apply an ice bag, or Leiter's coil of lead pipe,

*Paper delivered before Pennsylvania State Medical Society Meeting, in Philadelphia, May 17, 1894.

around the ear, with ice water flowing through it. If these mild measures are insufficient, powders of bismuth with morphia may be used, grading them according to the age of the child, with the use of the artificial leech or very small cups.

Acute middle ear catarrhal attacks in children, commencing in the nose, and extending through the Eustachian tube to the middle ear, producing deafness, should be carefully treated by the relief of pain, and even, if necessary, puncturing the membrana tympani —cleansing by a mild antiseptic solution or powder, followed by constitutional remedies adapted to the peculiar constitution of the child.

The throat must be attended to by applications to the naso-pharyngeal mucous membrane.

If this middle ear disease is not promptly treated, by opening the Eustachian tube, the disease is apt to involve the mastoid cells, especially the superficial layer. The following is the method of inflating the Eustachian tube in young children: We inflate the nose, and listen for the thud, normal, or abnormal moist sounds—these latter will make the diagnosis certain. The air douche operation of Politzer consists in a condensation of the air in the naso-pharynx, by a strong inflation into the cavity, while the nostrils are closed with the fingers. In the case of adults, it is necessary they should at the same time swallow, in order that the raised palate may close the naso-pharynx behind, and also because the act of swallowing with the mouth shut, opens the Eustachian tube, and thus furnishes a passage of air into the middle ear. In children, however, the swallowing is not absolutely necessary, because the naso-pharynx is so small that the condensation of the air is greater than in adults, and because the tubes also, in children, are relatively wider than in adults, and the action of the compressed air can, therefore, more readily reach the ear.

When this method of inflation is to be employed, instead of the rubber bag for air, as is generally used, with hard, long nozzle to be inserted back into the nose, a short rubber tube is what I use, the two ends of which are furnished with a quill, bone or ivory termination—one for the mouth of the physician, mother or nurse, and the other for the nose of the patient. After cleansing the nose by gently blowing, washing or wiping it out, the end is inserted and kept in place with the finger and thumb, and with the other end a blast of warm air is blown into the middle ear. At first the little patient is frightened, and grasps at the instrument and the ear; but after a time it gradually becomes accustomed to it, as it gives no real pain, and it rather becomes a source of amusement. Any intelligent mother or nurse could practice this operation when so ordered.

The physician must be sure that the cause of deafness is an exudation of pus, mucous or serum into the middle ear. If the child has had an earache, and there has been perforation of the membrana shown by a discharge, the simple act of blowing the nose will open the tube; this should always be taught a child, unless too young, when the nose must be cleansed several times a day with a soft camel's hair brush or small syringe and warm water, with a few grains of bicarbonate of soda. If, however, there has been inflammation of the middle ear of the child, the result of a cold from naso-pharyngeal catarrh, acute exanthema or pneumonia, attended with pain in the ear, or if the acute symptoms have been relieved by leeching, natural or artificial, hot or ice water applications, chloroform vapor, etc., the child recovers.

Frequently, from neglect, the child becomes absolutely deaf. Such a case was presented for treatment only yesterday. A child four years of age, completely deaf, never having been treated before; the cause could not be given by either the parents or the physician who brought it. It was not a deaf mute, as the child had a strong voice.

This is the reason that the statistics from the so-called deaf mute institutions are of so little value. The cause of each case should be carefully investigated and a correct diagnosis be given by the physician in charge, not left to the superintendent, who is not always a medical man.—*Laurence Turnbull, M.D., Philadelphia, Pa., in Virginia Medical Monthly.*

